# Transmembrane-4 L-Six Family Member-1 Is Essential for Embryonic Blood Vessel Development

**DOI:** 10.3390/cimb46110781

**Published:** 2024-11-18

**Authors:** Chi-Iou Lin, Anne Merley, Hiromi Wada, Jianwei Zheng, Shou-Ching S. Jaminet

**Affiliations:** 1 Center for Vascular Biology Research, Department of Pathology, Beth Israel Deaconess Medical Center, Harvard Medical School, Boston, MA 02215, USA; clin@riverview.org (C.-I.L.); anne_merley@brown.edu (A.M.); hwada@ric.u-tokyo.ac.jp (H.W.); zhengjianwei@bjtth.org (J.Z.); 2 Anesthesiology Department, Riverview Hospital, Noblesville, IN 46060, USA; 3 Center for Animal Resources and Education, Brown University, Providence, RI 02912, USA; 4 Isotope Science Center, The University of Tokyo, Tokyo 113-0032, Japan; 5 Department of General Surgery, TianTan Hospital, Capital Medical University, Beijing 100070, China; 6 Angiex Inc., Cambridge, MA 02140, USA

**Keywords:** TM4SF1, endothelial cell, mesenchymal stem cell, knockout mice, blood vessel development, intraventricular and subarachnoid hemorrhage

## Abstract

Transmembrane-4 L-six family member-1 (TM4SF1) is a small cell surface glycoprotein that is highly and selectively expressed on endothelial cell and mesenchymal stem cell surfaces. TM4SF1 regulates cellular functions by forming protein complexes called TMED (TM4SF1-enriched microdomains) that either recruit growth-factor activated proteins and internalize them via microtubules to distribute the recruited molecules intracellularly or support the formation of nanopodia for intercellular interactions extracellularly. Through a genetically manipulated mouse model for global *Tm4sf1* gene knockout, we demonstrate here that TM4SF1 is essential for blood vessel development. *Tm4sf1*-null embryos fail to develop blood vessels and experience lethality at E9.5. *Tm4SF1*-heterozygous embryos are smaller in body size during early embryonic development, and almost half die in utero due to intracranial hemorrhage in the intraventricular and subarachnoid space, which becomes apparent by E17.5. Surviving *Tm4SF1*-heterozygotes do not display overt phenotypic differences relative to wild type littermates postnatally. Together, these studies demonstrate that TM4SF1, through its molecular facilitator and nanopodia formation roles in TMED, intimately regulates blood vessel formation during embryonic development.

## 1. Introduction

Transmembrane-4 L-six family member-1 (TM4SF1) is a small plasma membrane glycoprotein of tetraspanin topology but distinct homology from the thirty-three genuine tetraspanins [1,2]. TM4SF1 was originally discovered as a tumor-associated antigen [3,4] that is also expressed at low levels by a normal vascular endothelium [5,6,7]. Subsequently, we demonstrated that TM4SF1 is expressed at high levels in cultured endothelial cells originating from blood or lymphatic vessels [6,8], endothelial progenitor cells, and in bone marrow-derived mesenchymal stem cells [9], as well as in vivo in an endothelium undergoing pathological angiogenesis in both experimental models of angiogenesis in mice [6] and in human cancers [6,7]. Our studies further demonstrated that the depletion of TM4SF1 in endothelial cells through knockdown (i) prevents the cellular polarization necessary for endothelial cell migration and proliferation in vitro [6,8]; (ii) blocks intercellular interactions and junction formation in cultured endothelial cells [8]; and (iii) inhibits vessel maturation in pathological angiogenesis in vivo [6]. 

TM4SF1 regulates biological and cellular activities through its ability to form 100–300 nm diameter protein complexes called TMEDs (TM4SF1-enriched microdomains) on the surfaces of cells that express high levels of TM4SF1 in vitro [6,7,8,9] and the tumor endothelium of human cancers in vivo [7]. Each TMED contains three to fourteen TM4SF1 molecules and functions as a membrane dock that recruits membrane proteins and brings along their intracellular membrane-proximal signaling components [7,8]. Through a novel and as of yet unelucidated mechanism, TMEDs internalize from the cell surface via microtubules with an ultimate destination in the nuclear compartment [7] and distribute recruited proteins from the plasma membrane to intracellular locations. TMEDs also facilitate the adhesion of endothelial cells to the matrix by recruiting integrin-α5β1 [6], a process that enables the formation of nanopodia: thin, long, membrane-lined channels within which actin microfilaments extend and/or retract during cell movement, and which facilitate the homotypic and heterotypic intercellular interactions for stable vessel formation [6,8,9,10]. 

In this study, we investigated TM4SF1’s functional role in normal blood vessel development using a global knockout strategy in mice. We report that *Tm4sf1*-null embryos are avascular and experience lethality at E9.5, while *Tm4sf1*-heterozygous embryos are smaller in body size during early embryonic development, and about half die in utero due to an intracranial hemorrhage, which becomes apparent in the intraventricular and subarachnoid space by E17.5. Together these studies reveal that TM4SF1, through its ability to form TMED, intimately regulates blood vessel formation during embryonic development.

## 2. Materials and Methods

### 2.1. Anti-TM4SF1 Antibodies 

Anti-TM4SF1 antibodies were the same as used in our earlier studies: (1) the anti-mouse TM4SF1 antibody 2A7A (a chimeric antibody with the variable region of a rabbit anti-mouse TM4SF1 monoclonal antibody and the constant region of a human IgG1) [11] and (2) the anti-human TM4SF1 antibody 8G4 [IgG1, mouse monoclonal [9,10]; MilliporeSigma (Burlington, MA, USA), catalog number MABC1723].

### 2.2. Cells and Cell Culture

All seven human primary endothelial cells (ECs), HUVEC (human umbilical vein EC), HLMEC (lung microvascular EC), HDMEC, (human dermal microvascular EC), HCAEC (human coronary artery EC), HPAEC (human pulmonary artery EC), ECFCs (endothelial colony forming cells), HMVEC-dLy (human dermal microvascular lymphatic EC), as well as HBdSMC (smooth muscle cells, bladder), HDF (fibroblast, dermal), HemaLP (epidermal melanocytes), and bone marrow-derived mesenchymal stem cells were purchased along with their respective media from Lonza (Walkersville, MD, USA), and used within six passages in all experiments conducted in this study. Three cell lines, 3T3 (fibroblast, mouse embryo), HEK393 (epithelial cells, kidney), and MS1 (mouse SV1 immortalized islet EC), were acquired from ATCC (American Type Culture Collection, Gaithersburg, MD, USA), cultured in their respective media suggested by ATCC, and used within five passages after being awakened from liquid nitrogen. Total RNAs for mouse leukocytes were extracted from fresh mouse blood while it was purchased from Thermo Fisher (Waltham, MA, USA) for human leukocytes.

### 2.3. Flow Cytometry

Experimental procedures were described in detail previously [10]. Briefly, 1 × 10^6^ freshly harvested cells (HPAEC, HDF, and HEK293) were washed in cold PBS, suspended in 1 mL of cold blocking buffer (PBS/2% FBS) that contained 100 ng of 1st antibody 8G4 or mouse IgG1 (Sigma, Saint Louis, MO, USA), and incubated at 4 °C for 30 min with occasional agitation. Cells were centrifuged (500× *g* for 5 min), followed by 100 ng/mL 2nd antibody (Alexa-488 labeled donkey anti-human or mouse IgG; Thermo Fisher) for another 30 min at 4 °C with washing 3× with cold PBS in between the incubations. Cell suspensions were analyzed with FACScan (Becton Dickinson, San Jose, CA, USA). A total of 10^4^ events were collected for each analysis. 

### 2.4. Generation of TM4SF1 Knockout Mice

Four key steps were involved to generate the *Tm4sf1* knockout mice: (1) we produced the linearized *Tm4sf1* genome targeting construct pM253/PUH-PDH/Neo (8461 bp), (2) introduced the construct into embryonic stem cells (ESCs) as described below, (3) identified mouse W4/129S6 ESCs that had *Tm4sf1* genome Ex3 to Ex5, targeted and replaced with loxP-Neo-loxP through homologous recombination, and (4) implanted the ESC clones into Fv129 mice (Charles River Laboratory, Shrewsbury, MA, USA). A schematic summary of the generation of the knockout mice is detailed in Supplementary Figure S2. All PCR primers used in *Tm4sf1* knockout mice are listed in Supplementary material Table S1. The Institutional Animal Care and Use Committee at Beth Israel Deaconess Medical Center, Boston approved all animal experiments (protocol #100-2011 and 111-2014).

RP24-402K15 chloramphenicol resistant Bac clone that contains mouse *Tm4sf1* locus (acquired from NIH) was used as the source of the mouse *Tm4sf1* genomic sequence. Four constructs were made to generate a *Tm4sf1* gene targeting construct (Supplementary Figure S2): (**i**) **pM253/UH-DH (5.7 kb) plasmid**. PCR amplified UH (657 bp; 5′ Ex3 flanking region) and DH (634 bp; reside in intron 6) DNA fragments from RP24-402K15, cut with Xba1 and ligated to generate UH-DH, followed by Kpn1 and HindIII, which were cut for insertion into the pM253 vector (provided by Dr. W.C. Aird [12,13]) to produce pM253/UH-DH (ampicillin resistant). (**ii**) **pM253/UH-HR-DH (13.5 kb) gene repair vector**. pM253/UH-DH plasmid was linearized and UH-DH arms separated through an Xba1 cut and electroporated into RP24-402K15/EL-250 bacteria for homologous recombination (HR) to bring in *Tm4sf1* Ex3 to Ex6 (9013 bp) genome segment to the plasmid. The plasmid was confirmed via PCR through UP and DP primer sets. (**iii**) **PUH/loxP-Neo-loxP/PDH (2.85 kb) cassette**. PCR amplified the PUH (463 bp; 5′ Ex3 flanking region adjacent to Ex3) and PDH (587 bp; 3′ Ex5 intron region) DNA fragments from RP24-402K15, respectively; they were cut with Bsa1 and BsmB1 and inserted on either side of the loxP-Neo-loxP cassette. (**iv**) **pM253/PUH/loxP-Neo-loxP/PDH (12.9 kb) targeting plasmid**. RP24-402K15/EL-250 bacteria that contain pM253/UH-HR-DH gene repair vector were transfected with PUH/loxP-Neo-loxP/PDH cassette to generate pM253/PUH/loxP-Neo-loxP/PDH plasmid through HR (neomycin resistant). The removal of 2345 bp long Ex3 to Ex5 was then confirmed via PCR using TM-UPH-Neo forward and reverse primers. The 8461 bp targeting construct was then released via Kpn1 and HindIII, cut and transfected W4/129S6 ESCs (Taconic Biosciences, Albany, NY, USA). G418 antibiotic (Thermo Fisher) selection at 350 ug/mL was initiated 24 h following targeting and neomycin-resistant ESC clones were screened for successful homologous recombination with PCR using the genotyping primer sets. 

Positive *Tm4sf1* −/+ ES clones were amplified and submitted to the BIDMC transgenic core facility for injection into blastocysts. Briefly, targeted ESC clones were microinjected into C57BL/6-derived blastocyst stage embryos which were then transplanted into the uteri of recipient C57BL/6 females (Charles River Laboratory) to generate chimeric mice. Resulting chimeric males were bred with C57BL/6 females to obtain mice heterozygous for the inactivated loci. The heterozygous F1 male were back-crossed with C57BL/6 females to generate the F2 heterozygous male and repeated for 8 generations to stabilize the chromosome before conducting the *Tm4sf1*-heterozygotes male and female inbred mating to obtain homozygous embryos. 

### 2.5. PCR Genotyping

Genotyping was conducted using chromosomal DNA extracted from either yolk sac or a 2 mm tail clip from P14 to P21 postnatal mice. PCR analyses were performed with primers specific for the wild type and targeted alleles. Tissues were submerged in Gitschier Buffer in the presence of β-mercaptoethanol and proteinase-K to release chromosomal DNA. PCR conditions as described by the manufacturer of AmpliTaq Polymerase (Perkin-Elmer Cetus, Norwalk, CT, USA) were used to generate fragments from wild type (596 bp) and targeted (397 bp) alleles on 1% agarose gels. 

### 2.6. Multi-Gene Transcriptional Profiling (MGTP) Approach to Quantitative Real-Time PCR (qPCR)

MGTP is a qPCR technique that efficiently quantifies mRNA copies per cell [14] and is performed by MGTP core. Total RNA was prepared using the RNeasy kit with DNase-I treatment (Qiagen, Chatsworth, CA, USA) and cDNA using random primers and SuperScript III (Thermo Fisher). Total RNAs for both a human aorta and vein were purchased from BioChain (Newark, CA, USA). For each data point, mean ± SD were calculated from three different samples from three separate experiments. PCR reactions for each cDNA sample were performed in duplicate. Transcript abundances were normalized/10^6^ 18S-rRNA copies to an approximate number of transcripts/cell [14]. All MGTP primer sequences used in the study are listed in Supplementary Table S2. 

### 2.7. Mouse Organ Harvest and Immunohistochemical (IHC) Staining

Experimental procedures were described in detail in previous studies [15]. Briefly, mouse tissues were immediately placed in 4% paraformaldehyde (PFA) after harvesting from 8 week old C57BL/6 mice, and cryostat sections were immunostained with anti-mouse TM4SF1 antibody 2A7A followed by donkey anti-human HRP-conjugated secondary antibodies (Thermo Fisher). Normal human liver section was prepared from surgically removed liver transplant by Dr. Lawrence Brown at BIDMC’s Pathology Dept and placed in 4% paraformaldehyde followed by OCT mounting and cryostat sectioning for staining with mouse anti-human TM4SF1 antibody 8G4, with subsequent anti-mouse HRP-conjugated 2nd antibodies (Thermo Fisher). All IHC images were representative selections from at least three separate sections and were taken by Dr. Harold Dvorak. For wholemount mouse embryo and retina staining, Alexa488-conjugated 2A7A was used and images were captured via Keyence BZ-9000E microscope (Itasca, IL, USA).

### 2.8. Embryo Images, Videos and Immunostaining

All bright field images or videos of embryos harvested from different developmental stages were taken from live cam attached Wild Photo Mikroskop M400 stereo photomicroscope (Martin Microscope, Easley, SC, USA). Wholemount staining was carried out as described [12]. Briefly, E9.5 embryos were fixed for 4 h in 4% PFA in PBS at 4 °C, rinsed in PBST (0.1% Tween-20/PBS), and incubated with Alexa488-conjugated anti-mouse TM4SF1 antibody 2A7A in blocking buffer (PBST/2% FBS) for two nights at 4 °C. Fluorescence images were then acquired via Keyence microscope after thoroughly washing the embryos in PBST overnight at 4 °C. For *Tm4s1*-histology staining, 4% PFA fixed embryos were cryoprotected overnight in 20% sucrose/PBS, embedded in OCT (Tissue-Tek), sectioned (6 μm), and stained with the 2A7A antibody, followed by HRP-conjugated anti-human 2nd antibody and Eosin counterstaining. For H&E staining, embryos were preserved in 50% ethanol before being processed for FFPE for H&E staining at the BIDMC’s tissue processing core. 

### 2.9. Statistical Analysis

Each experiment was repeated at least three times and the statistical difference between groups is presented as mean ± standard deviation. Analysis was performed using GraphPad Prism 10.0 software. The significance of differences between groups was assessed by Student’s *t*-test; *p* values < 0.05 were considered statistically significant.

## 3. Results 

### 3.1. TM4SF1 Expression Is Largely Limited to Endothelial Cells In Vitro and In Vivo

We surveyed TM4SF1 expression in cultured endothelial cells and six non-endothelial cell and non-tumor cell types that originated from both human and mouse tissues. qPCR revealed that human TM4SF1 expression in non-endothelial cells, with the exception of mesenchymal stem cells (MSCs, bone marrow derived), was either very low as in smooth muscle cells (HBdSMCs, bladder) and fibroblasts (HDF, dermal), or was not detectably expressed as in kidney epithelial cells (HEK293), epidermal melanocytes (HemaLP), and white blood cells (WBCs) (Supplementary Figure S1). TM4SF1 expression in cells of mouse origin such as MS1 (SV40-immortalized mouse islet endothelial cells), 3T3 (embryonic fibroblasts), and mWBCs was consonant with TM4SF1 expression in the corresponding human cell lineages (Supplementary Figure S1A). 

**Figure 1:**
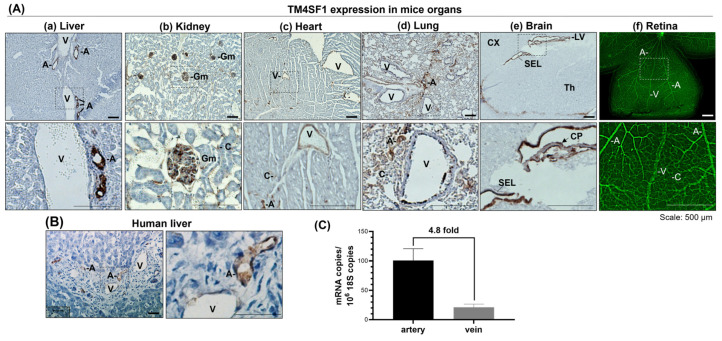
TM4SF1 expression in the endothelium *in vivo*. TM4SF1 IHC staining with HRP-conjugated 2nd antibody and eosin was conducted in tissue sections prepared from (**A**) 8 week old C57BL/6 mouse organs [liver (**a**), kidney (**b**), heart (**c**), lung (**d**), and brain (**e**)] and (**B**) human liver. Wholemount staining was performed in postnatal day 18 C57BL/6 mouse retina (**f**) with Alexa488-conjugated anti-mouse TM4SF1 antibody 2A7A. Representative IHC staining in both (**A**,**B**) tissue sections showed that TM4SF1 expression was largely limited to blood vessels with a tendency for the highest TM4SF1 expression to be in arteries (A), intermediate expression in veins (V), and the lowest expression in capillaries (C). Positive TM4SF1 expression was also seen in kidney glomeruli (Gm) and in the choroid plexus (CP; black arrow), the epithelium of lateral ventricles (LV), along with the subependymal cell layer (SEL) in the brain; no expression was observed in the cortex (CX) and thalamus (Th). (**C**) qPCR using total RNA (normalized against 10^6^ 18S copies) acquired from three different human aorta and pulmonary vein samples showed that TM4SF1 expression was 4.8-fold higher in the artery than in the vein (*p* = 0.0026).

IHC staining in tissue sections prepared from six different mouse tissues (liver, lung, heart, kidney, brain, and retina) provided further evidence that TM4SF1 expression was limited to the vascular endothelium, and that expression was highest in the endothelium of arteries, followed by veins and capillaries (Figure 1A). Strong TM4SF1 expression is also seen in kidney glomeruli and in the choroid plexus epithelium of the lateral ventricle as well as the subependymal cell layer in the mouse brain (Figure 1A). Similar TM4SF1 localization was demonstrated in both mouse and human tissues; for example, the human liver exhibits arterial > venous > capillary endothelial staining (Figure 1B). In agreement, qPCR demonstrated that TM4SF1 gene expression in RNA from the human aorta was 4.8-fold greater than that from the pulmonary vein (*p* = 0.0026; Figure 1C).

### 3.2. Tm4sf1-Knockout Mice Are Embryonic Lethal at Embryonic Day 9.5 (E9.5) 

The mouse *Tm4sf1* gene is encoded on the minus strand of chromosome-3 from nucleotides 57,105,910 to 57,089,531 and contains seven exons (Ex). The protein coding region begins in Ex3 and ends in Ex7 and encodes 202 amino acids (Supplementary Figure S2A). We used standard gene targeting strategies to generate *Tm4sf1*-knockout mice; *Tm4sf1*’s first three coding exons (Ex3 to Ex5) start with an initiation codon ATG, and our targeting strategy was designed to completely eliminate TM4SF1 protein translation by deleting Ex3 to EX5 (Supplementary Figure S2B). 

Genotypes of progeny of intercrossed *Tm4sf1*-heterozygous mice from E9.5 to weaning age (WA) (postnatal day 14 to day 21) are presented in Table 1. At WA, 154 pups were accounted to 30 litters, 74 pups were heterozygous (het; +/−), 80 were wild type (wt; +/+), and zero were knockout (ko; −/−) (Table 1). The ratio of +/− to +/+ was 0.93 at WA, substantially different from the expected Mendelian ratio of 2. The 5.1 pups/litter was also below the normal average of 8 pups/litter (the normal distribution for wt:het:ko for an eight pup litter would be 2:4:2). These results indicate that all *Tm4sf1*-knockout mice died in utero; of the *Tm4sf1*-heterozygous, (1) working from the expected litter size, only 62% were born live (74 pups out of an expected 120 pups for the total of 30 litters); or alternatively, (2) working from Mendelian ratios, about 48% (74 pups out of 154 pups for the total of 30 litters with wt:het ratio of 0.93 instead of 2.0).

**Figure 2:**
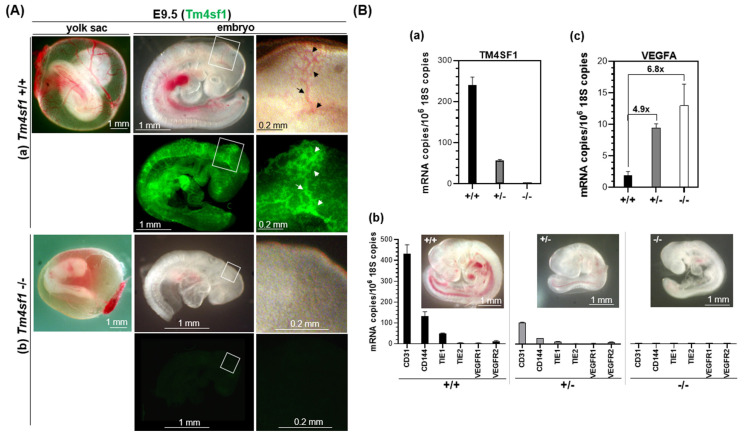
*Tm4sf1*-knockout mice are embryonically lethal at E9.5. (**A**) Representative E9.5 bright field embryo images of littermates show developing blood vessels in both the yolk sac and embryo in *Tm4sf1* +/+ (**a**) but not in *Tm4sf1* −/− (**b**). Wholemount embryo staining using Alexa488-conjugated 2A7A demonstrated TM4SF1 protein localization in the *Tm4sf1* +/+ embryo vasculature (white arrows indicate the same locations as the black arrows in bright field images), but no 2A7A staining occurred in the *Tm4sf1* −/− embryo. (**B**) qPCR was conducted on total RNA prepared from E9.5 littermate embryos (three each of +/+, +/−, and −/− from three different litters). (**a**) Representative RNA quantification demonstrated that *Tm4sf1* −/− embryos, unlike *Tm4sf1* +/+ and +/− embryos, did not express the *Tm4sf1* gene and that (**b**) the known vascular markers CD31, CD144, TIE1, TIE2, VEGFR1, and VEGFR2 were also too low to be detected; and, (**c**) in comparison to *Tm4sf1* +/+ embryos, VEGFA expression was 6.8-fold (*p* = 0.00003) higher in *Tm4sf1* −/− embryos and 4.9-fold (*p* = 0.0014) higher in *Tm4sf1* +/− embryos. (**b**) The representative inset littermate embryo images show an absence of blood vessels in the −/− embryos, and a small body size in the +/− embryo in comparison to the +/+.

Although only 48–62% of the *Tm4sf1*-heterozygous reached WA, a near Mendelian ratio of 1:2:1 (wt:het:ko) was observed at E9.5 to E12.5 (Table 1). However, none of the homozygous *Tm4sf1*-null embryos were viable with a vital heartbeat; see representative movies taken from E9.5 *Tm4sf1*-wild type (Supplementary Movie S1) and *Tm4sf1*-knockout (Supplementary Movie S2) littermate embryos. Representative E9.5 embryos further demonstrated that unlike *Tm4sf1*-wild type embryos (Figure 2A(a)), *Tm4sf1*-knockout embryos lacked visible blood vessels in the yolk sac and embryo body in bright field images and lacked TM4SF1 positively stained blood vessels in immunofluorescence wholemount (Figure 2A(b)). E10.5 littermates provided further evidence of the inability of *Tm4sf1*-knockout embryos to generate vasculature (Supplementary Figure S3). A representative mid-sagittal section of a wild type E10.5 embryo revealed that all major vessels stained positively for TM4SF1, as did the cephalic mesenchyme, the condensing mesenchyme in the head (Supplementary Figure S4).

In accordance with yolk sac genotyping, the qPCR performed on the total RNA extracted from E9.5 embryos demonstrated the absence of detectable *Tm4sf1* gene expression in the *Tm4sf1*-knockout embryos (Figure 2B(a)). *Tm4sf1*-knockout embryos also were deficient in blood vessel markers including CD31, CD144, TIE1, TIE2, VEGFR1, and VEGFR2 (Figure 2B(b)). Expression of TM4SF1 and the six vascular markers in *Tm4sf1*-heterozygous embryos was less than half of that in their *Tm4sf1*-wild type littermates (Figure 2B(b)). These differences in vascular gene expression were accompanied by differences in embryo phenotype: representative E9.5 embryo images from the same litter showed that the *Tm4sf1*-heterozygous embryo exhibited a smaller body size than their wild type littermates, and the *Tm4sf1*-knockout embryo lacked blood vessels (Figure 2B(b), embryo image inset). VEGFA expression was, respectively, 6.8-fold and 4.9-fold higher in the *Tm4sf1*-knockout and *Tm4sf1*-heterozygous embryos than in wild type embryos (Figure 2B(c)).

### 3.3. Tm4sf1-Heterozygous Embryos Were Smaller in Size, and Approximately Half Evolved Significant Brain Hemorrhage

**Figure 3:**
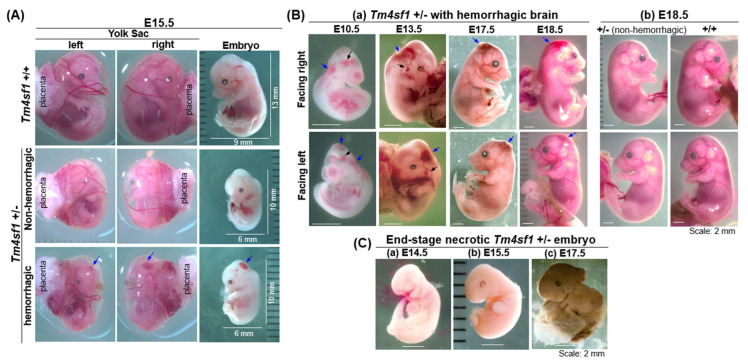
*Tm4sf1*-heterozygous embryos can develop lethal brain hemorrhage. Representative images from the sequential developmental stages of mouse embryos portray brain hemorrhages that occurred in some *Tm4sf1* +/− embryos. (**A**) Two yolk sac images were taken with the placenta positioned at left or right. E14.5 *Tm4sf1* +/− embryos had a smaller body size than their wild type littermate, and one of the two *Tm4sf1* +/− embryos displayed hemorrhage in the head (blue arrows). No obvious hemorrhage appeared in other parts of the embryo body including the yolk sac. (**B**) The hemorrhage can be seen as early as E10.5 and became more apparent over the subsequent days of development (blue arrows), with some embryos also showing a vascular defect near the jugular vein (black arrows). Brain hemorrhages did not affect the embryo growth rate; at E18.5, *Tm4sf1* +/− embryos with and without brain hemorrhage were comparable in body size and had nearly caught up in size to *Tm4sf1* +/+ embryos. (**C**) Deceased *Tm4sf1* +/− embryos were noted during embryo harvests at (**a**) E14.5, (**b**) E15.5, and (**c**) E17.5.

Abnormalities in *Tm4sf1*-heterozygous embryos were clearly noted starting at about E14.5, when many developed brain hemorrhage (Figure 3; Table 1). Representative E15.5 littermate embryo images revealed that *Tm4sf1*-heterozygous displayed a smaller body size than their wild type littermate, with one of the two *Tm4sf1*-heterozygous exhibiting brain hemorrhage (Figure 3A, blue arrow), while other regions of the body, including the yolk sac, appeared to be normal.

Representative images of developing embryos provide further evidence that visible vascular defects in *Tm4sf1*-heterozygous embryos were largely confined to the head (Figure 3B, blue arrow) with some also seen in the vicinity of the jugular vein (Figure 3B, black arrows). Occasionally, some remnants of dead *Tm4sf1*-heterozygous embryos were identified during embryo harvest (Figure 3C). This is consistent with the progressively declining ratio of non-hemorrhagic *Tm4sf1*-heterozygous to wild type embryos from the expected 2:1 Mendelian ratio and observed as 1.16 at E14.5, 0.91 at E18.5, and 0.93 at WA. These data imply that lethal vascular defects can happen before E18.5 in *Tm4sf1*-heterozygous embryos.

**Figure 4:**
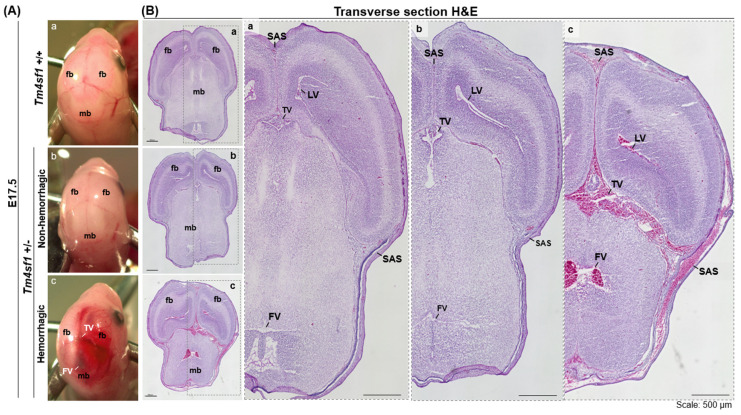
Brain hemorrhage in some *Tm4sf1* +/− embryos. Representative bright field head images of E17.5 *Tm4sf1* +/+ and +/− littermates are shown in (**A**) and their respective transverse H&E histological staining are shown in (**B**). (**A**) A lack of integrity in the fb (forebrain)-fb and fb-mb (midbrain) junctions and an accumulation of blood around the TV (third ventricle) and FV (fourth ventricle) were seen in the hemorrhagic *Tm4sf1* +/− embryo (**c**) but not in the non-hemorrhagic *Tm4sf1* +/− (**b**) and the *Tm4sf1* +/+ (**a**) embryos. (**B**) All four ventricles, TV, FV, and left and right LV (lateral ventricle), and the SAS (subarachnoid spaces) were filled with blood in the hemorrhagic *Tm4sf1* +/− embryo (**c**) whereas all structures appeared normal in both the non-hemorrhagic *Tm4sf1* +/− (**b**) and the *Tm4sf1* +/+ (**a**) embryos.

A higher resolution bright field image of a representative E17.5 *Tm4sf1*-heterozygous embryo that exhibited brain hemorrhage demonstrates a lack of integrity in the forebrain–forebrain (fb-fb) and forebrain–midbrain (fb-mb) junctions and an accumulation of blood around the third and fourth ventricles (Figure 4A(c)). The transverse H&E section of the hemorrhagic *Tm4sf1*-heterozygous embryo showed that all four ventricles (lateral left and right, third, and fourth) in the head along with the subarachnoid space were filled with blood (Figure 4B(c)), but no hemorrhage was observed in the cortex (Figure 4B(c)). The *Tm4sf1*-heterozygous littermate without brain hemorrhage resembled wild type embryos and showed normal integrity in the fb-fb and fb-mb regions (Figure 4A(a,b)), without blood accumulation in the ventricles or subarachnoid space (Figure 4B(a,b)).

Whether or not brain hemorrhage was experienced, *Tm4sf1*-heterozygous embryos were smaller in body size at E15.5, but progressively caught up with wild type embryos at later ages and showed minimal difference in body size at the time of birth (Figure 3B(b); Supplementary Figure S5A). Tracking postnatal growth via body weight also demonstrated that *Tm4sf1*-heterozygous and wild type had a similar growth rate (Supplementary Figure S5B), and *Tm4sf1*-heterozygous mice also experienced normal fertility and lifespan. The ratio of live born males to females in the wild type and in *Tm4sf1*-heterozygous litters were 0.95 and 0.9, respectively (Supplementary Figure S5C). Overall, the results suggest that the challenging period for mice with deficient TM4SF1 protein expression is during embryonic development, and lethal embryonic brain hemorrhages are equally likely to occur in male and female *Tm4sf1*-heterozygous embryos.

## 4. Discussion

Through genetic manipulation approaches, we demonstrate here that *Tm4sf1*-null mice experience embryonic lethality by E9.5 due to a failure to form blood vessels, and that the suboptimal level of TM4SF1 in *Tm4sf1*-heterozygotes causes a smaller body size during early embryonic development with almost half of the embryos experiencing a lethal vascular defect in the head that led to intraventricular and subarachnoid hemorrhage.

Differential expression of TM4SF1 in different vascular beds was observed in vivo, with arteries having the highest expression, veins intermediate, and capillaries the lowest (Figure 1). This result was unanticipated since cultured endothelial cells, regardless of their in vivo bed of origin, showed uniformly high and similar levels of TM4SF1 expression in vitro (Supplementary Figure S1). We presume that this differential expression in vivo is the result of endothelial cell interactions with accessory cells to generate distinct vascular beds [16,17], a process supported by mesenchymal stem cell differentiation to smooth muscle cells for the formation of larger vessels like arteries or to pericytes for the support of veins and capillaries [18,19]. We exclude the potential involvement of shear stresses as our studies applying laminar flow (steady, non-turbulent) to cultured endothelial cells via an orbital shaker did not affect TM4SF1 level in our current ongoing studies.

VEGF-A plays a critical role in endothelial cell proliferation for vasculogenesis and angiogenesis through the activation of its receptors expressed on the cell surface [20,21,22]. Our prior studies demonstrated that, although VEGF-A was supplied in abundance, the depletion of TM4SF1 through knockdown [6] resulted in (i) an inability of endothelial cells to perform cytokinesis for proliferation, polarization for movement, or intercellular interactions in vitro; and (ii) the inhibition of blood vessel maturation in a mouse model of VEGF-A provoked pathological angiogenesis in vivo. In a similar manner, high VEGF-A expression failed to avert the avascular nature of E9.5 *Tm4sf1*-null embryos (Figure 2). The high VEGF-A expression seen in E9.5 *Tm4sf1*-heterozygous embryos, perhaps caused by hypoxia [23] for urging blood vessel development and conceivably originated from the choroid plexus, ventricular neuroectoderm cells, head mesenchyme, and between the somites [24,25,26], may have contributed to the ultimate recovery of normal body size in the heterozygous embryos over the course of developmental time (Figure 3B; Supplementary Figure S5). The smaller embryo body size observed during the *Tm4sf1*-heterozygotes’ development is not uncommon among the knockout mice in others studies, in which the target gene is involved in blood vessel development; examples include *integrin-αvβ8* [27], *Erg* transcription factor [13], and proteins in the VEGF signaling cascade [25,28].

The circulatory system is the first and most essential organ system to begin functioning during embryonic development and is assembled through vasculogenesis and angiogenesis followed by vascular remodeling via the recruitment of vascular supporting accessory cells [29,30]. In mouse embryos, the initial establishment of vasculogenesis that gives rise to the first primitive vessels in the yolk sac occurs at E6.5; establishment of the cardiovascular system commences at E7, starting with the development of the endocardium [31]. The umbilical artery, the site of hematopoetic stem cell development [32], is initiated in the allantois at E7.5 and fused to the chorion at E8.5 to support the morphogenetic process of vascular remodeling and the circulation to and from the yolk sac and heart in the embryo proper [31,33]. Impairments in any of those steps, for example in mice with genetically manipulated VEGF [25,26] or VEGF receptor signaling [34,35,36], will lead to embryonic lethality by E9.5.

Given that TM4SF1 protein expression is specific to the vascular endothelium and the mesenchyme in vivo (Figure 1; Supplementary Figure S4) and cultured cells in vitro (Supplementary Figure S1), TM4SF1 is likely to influence embryonic blood vessel development through both the endothelial cells and the mesenchymal stem cells. Endothelial cells express high levels of VEGFR2/Flk-1 and PDGF-β, and mesenchymal stem cells express high levels of the corresponding ligand VEGF-A and receptor PDGFRβ [9,37,38], an indication that intimate interactions between the two cell types facilitate blood vessel formation [16]. As unpublished studies with cultured endothelial cells show that PLCγ-1, the immediate downstream mediator of VEGFR2/Flk-1 signaling, which activates ERK upon Flk-1 activation to produce an embryonic lethal phenotype, similar to that of Flk-1 null mice [39], is recruited to TMED. PLCγ-1 is also a downstream mediator of PDGFR in mesenchymal stem cells [38]. We anticipate that a defect in PLCγ-1 functional activities may be one of the molecular mechanisms underlying the *Tm4sf1*-null phenotype. Furthermore, the insufficient TMED-mediated intracellular transport of recruited molecules and/or intercellular interactions in both endothelial cells and mesenchymal stem cells may contribute to the impairment of vasculogenesis, and to the impaired remodeling seen in *Tm4sf1*-heterozygous embryos. A detailed characterization of TMED complexes to identify their recruited proteins via mass spectrometry is currently underway.

A number of questions remain unanswered: why was hemorrhage largely limited to the head but no other parts of the *Tm4sf1*-heterozygous embryos? Also, why did hemorrhage happen in only about half of the *Tm4sf1*-heterozygotes, and not all? We noted that the TM4SF1 protein is highly expressed in the cephalic mesenchyme of E10.5 mouse embryos, with no noticeable expression in the cortical neuroectoderm (Supplementary Figure S3); TM4SF1 protein expression is also seen in the choroid plexus epithelium of the lateral ventricle and the subependymal cell layer in adult mouse brains (Figure 1). However, we currently do not have data to elucidate how TM4SF1 expression in these locations influenced the phenotypic development of *Tm4sf1*-heterozygotes. Specifically, we do not know whether the expression in cephalic mesenchyme is transitory, and if differences in TM4SF1 expression regulation in the mesenchyme contributed to the occurrence of vascular hemorrhage. We anticipate, based on our occasional observations of the end-stage necrosis of *Tm4sf1*-heterozygous embryos as early as E14.5 (Figure 4A), that some *Tm4sf1*-heterozygous embryos may experience lethality before they have developed the apparent intraventricular and subarachnoid hemorrhage seen near birth (Figure 4B). This implies considerable variability among *Tm4sf1*-heterozygous embryos, with some encountering more severe vascular defects with early lethality, others intraventricular and subarachnoid hemorrhage, or normal development.

E14.5 is the transition stage from the embryonic to fetal period and is considered to be the most important time point for developmental disorder analyses [40]. In many other perinatally or prenatally lethal mutant mouse lines, half of the embryos survive until E14.5, including some in which the cause of death is intracerebral hemorrhage by mutations that affect endothelial cell function such as *Fli1* [41] and *Erg* [13] transcription factors, and *integrin-αvβ8* adhesion molecules [27,42]. Nonetheless, embryonic intraventricular and subarachnoid hemorrhage has not been reported in studies using genetically manipulated mice, including manipulations through the VEGF signaling cascade [25,26,34,35]. Ventricles are a communicating network of chambers filled with cerebrospinal fluid that in mouse embryos, is predominantly produced in the lateral ventricles, transported through the ventricular system, and enters the subarachnoid space through the foramina at the fourth ventricle for final absorbance [43]. In mouse embryos, the lateral (I and II), third, and fourth ventricles develop around E10 and the cerebral aqueduct connecting the third and fourth ventricle is formed and becomes the narrowest part of the cerebrospinal fluid system during late embryonic brain development [43]. We infer that the vascular integrity required to support the ventricles for cerebrospinal fluid clearance is especially challenged by the suboptimal level of TM4SF1 expression.

Future studies, combining proteomic analysis for protein identification and tracing the fate of *Tm4sf1*-null cells via lineage tracing with spatial biology imaging techniques, possibly aided by the knock-in of a reporter gene like lacZ to the *Tm4sf1* locus or conditional *Tm4sf1*-knockout, will address where and when TM4SF1 is expressed during embryo development, and how it works with recruited proteins for the regulation of blood vessel formation and maturation.

## 5. Conclusions

We report that *Tm4sf1*-null embryos fail to form blood vessels and experience embryonic lethality at E9.5; *Tm4sf1*-heterozygotes are smaller in size than wild type embryos during early embryonic growth, and half die in utero, often with intraventricular and subarachnoid hemorrhage. The half of *Tm4sf1*-heterozygous pups that are born alive appear normal, fertile, and have no apparent postnatal developmental defects. As noted in our prior reports, TMED form on the cell surface when TM4SF1 is highly expressed and serve as critical signaling mediators through internalization via microtubules to the nucleus and through nanopodia formation for intercellular interactions. This study displays that the timeliness of an optimal level of TM4SF1 expression to support TMED formation for endothelial cell and mesenchymal stem cell signaling and intercellular interactions are of critical importance during embryonic blood vessel development.

## Figures and Tables

**Table 1:** Genotypes of progeny from *Tm4sf1*-heterozygous intercrosses.

	# of Embryos (% over Total #)									
Age	+/+	+/− ^a^	+/− ^b^	+/− ^c^	−/−	Total #	Litters	#/Litter	Viable −/−	+/− ^a^: +/+
**E9.5**	34 (29.6)	63 (54.8)	0 (0.0)	0 (0.0)	16 (13.9)	113	12	9.4	0	1.85
**E10.5**	22 (30.1)	41 (56.2)	2 (0.0)	0 (0.0)	10 (13.7)	75	9	8.3	0	1.86
**E12.5**	33 (34.0)	58 (59.8)	0 (0.0)	0 (0.0)	6 (6.2) ^§^	97	10	9.7	0	1.76
**E14.5**	25 (39.1)	29 (45.3)	4 (6.3)	2 (3.1)	4 (6.3) ^§^	64	8	8.0	0	1.16
**E16.5**	27 (40.3)	26 (38.8)	7 (10.4)	1 (1.5)	6 (9.0) ^§^	67	8	8.4	0	0.96
**E18.5**	34 (39.1)	31 (35.6)	19 (21.8)	3 (3.4)	0 (0.0)	87	11	7.9	0	0.91
**WA**	80 (51.9)	74 (48.1)	0 (0.0)	0 (0.0)	0 (0.0)	154	30	5.1	0	0.93

**E**, embryonic day; **WA**, weaning age for genotyping; **a**, embryos without visible hemorrhagic blood vessel or live at the WA; **b**, embryos with visible hemorrhagic brain; **c**, necrotic dead embryo; **§**, embryos were deteriorating or being absorbed.

## Data Availability

The datasets used and/or analyzed during the current study are available from the corresponding author on reasonable request.

## Supplementary Materials

The following supporting information can be downloaded at: https://www.mdpi.com/article/10.3390/cimb46110781/s1. Figure S1: TM4SF1 expression in cultured cells *in vitro*; Figure S2: Genomic structure of *Tm4sf1* and targeting strategy to generate *Tm4sf1*-knockout mice; Figure S3: Defective in yolk sac vascular development in *Tm4sf1* knockout embryo; Figure S4: TM4SF1 protein expression in E10.5 wild type embryos; Figure S5: Growth of *Tm4sf1* +/+ and non-hemorrhagic +/− mice during prenatal and postnatal stages; Table S1: PCR primer sequences used in generating *Tm4sf1*-knockout mice; Table S2: Real-time PCR primer sequences used in MGTP approach to quantitative real-time PCR; Movie S1: TM4SF1 wt; Movie S2: TM4SF1 ko.

## References

[1] Wright M.D., Ni J., Rudy G.B. (2000). The L6 membrane proteins--a new four-transmembrane superfamily. Protein Sci..

[2] Stipp C.S., Kolesnikova T.V., Hemler M.E. (2003). Functional domains in tetraspanin proteins. Trends Biochem. Sci..

[3] Hellstrom I., Beaumier P.L., Hellstrom K.E. (1986). Antitumor effects of L6, an IgG2a antibody that reacts with most human carcinomas. Proc. Natl. Acad. Sci. USA.

[4] Marken J.S., Schieven G.L., Hellstrom I., Hellstrom K.E., Aruffo A. (1992). Cloning and expression of the tumor-associated antigen L6. Proc. Natl. Acad. Sci. USA.

[5] DeNardo S.J., O'Grady L.F., Macey D.J., Kroger L.A., DeNardo G.L., Lamborn K.R., Levy N.B., Mills S.L., Hellstrom I., Hellstrom K.E. (1991). Quantitative imaging of mouse L-6 monoclonal antibody in breast cancer patients to develop a therapeutic strategy. Int. J. Rad. Appl. Instrum. B.

[6] Shih S.C., Zukauskas A., Li D., Liu G., Ang L.H., Nagy J.A., Brown L.F., Dvorak H.F. (2009). The L6 protein TM4SF1 is critical for endothelial cell function and tumor angiogenesis. Cancer Res..

[7] Sciuto T.E., Merley A., Lin C.I., Richardson D., Liu Y., Li D., Dvorak A.M., Dvorak H.F., Jaminet S.C. (2015). Intracellular distribution of TM4SF1 and internalization of TM4SF1-antibody complex in vascular endothelial cells. Biochem. Biophys. Res. Commun..

[8] Zukauskas A., Merley A., Li D., Ang L.H., Sciuto T.E., Salman S., Dvorak A.M., Dvorak H.F., Jaminet S.C. (2011). TM4SF1: A tetraspanin-like protein necessary for nanopodia formation and endothelial cell migration. Angiogenesis.

[9] Lin C.I., Merley A., Sciuto T.E., Li D., Dvorak A.M., Melero-Martin J.M., Dvorak H.F., Jaminet S.C. (2014). TM4SF1: A new vascular therapeutic target in cancer. Angiogenesis.

[10] Lin C.I., Lau C.Y., Li D., Jaminet S.C. (2014). Nanopodia-Thin, fragile membrane projections with roles in cell movement and intercellular interactions. J. Vis. Exp. JoVE.

[11] Visintin A., Knowlton K., Tyminski E., Lin C.I., Zheng X., Marquette K., Jain S., Tchistiakova L., Li D., O’Donnell C.J. (2015). Novel Anti-TM4SF1 Antibody-Drug Conjugates with Activity against Tumor Cells and Tumor Vasculature. Mol. Cancer Ther..

[12] Yuan L., Janes L., Beeler D., Spokes K.C., Smith J., Li D., Jaminet S.C., Oettgen P., Aird W.C. (2013). Role of RNA splicing in mediating lineage-specific expression of the von Willebrand factor gene in the endothelium. Blood.

[13] Vijayaraj P., Le Bras A., Mitchell N., Kondo M., Juliao S., Wasserman M., Beeler D., Spokes K., Aird W.C., Baldwin H.S. (2012). Erg is a crucial regulator of endocardial-mesenchymal transformation during cardiac valve morphogenesis. Development.

[14] Wada Y., Li D., Merley A., Zukauskas A., Aird W.C., Dvorak H.F., Shih S.C. (2010). A multi-gene transcriptional profiling approach to the discovery of cell signature markers. Cytotechnology.

[15] Brown L.F., Dezube B.J., Tognazzi K., Dvorak H.F., Yancopoulos G.D. (2000). Expression of Tie1, Tie2, and angiopoietins 1, 2, and 4 in Kaposi’s sarcoma and cutaneous angiosarcoma. Am. J. Pathol..

[16] Swift M.R., Weinstein B.M. (2009). Arterial-venous specification during development. Circ. Res..

[17] Beck L., D’Amore P.A. (1997). Vascular development: Cellular and molecular regulation. FASEB J..

[18] Ding R., Darland D.C., Parmacek M.S., D’Amore P.A. (2004). Endothelial-mesenchymal interactions in vitro reveal molecular mechanisms of smooth muscle/pericyte differentiation. Stem Cells Dev..

[19] Gu W., Hong X., Le Bras A., Nowak W.N., Issa Bhaloo S., Deng J., Xie Y., Hu Y., Ruan X.Z., Xu Q. (2018). Smooth muscle cells differentiated from mesenchymal stem cells are regulated by microRNAs and suitable for vascular tissue grafts. J. Biol. Chem..

[20] Pettersson A., Nagy J.A., Brown L.F., Sundberg C., Morgan E., Jungles S., Carter R., Krieger J.E., Manseau E.J., Harvey V.S. (2000). Heterogeneity of the angiogenic response induced in different normal adult tissues by vascular permeability factor/vascular endothelial growth factor. Lab. Investig..

[21] Conway E.M., Collen D., Carmeliet P. (2001). Molecular mechanisms of blood vessel growth. Cardiovasc. Res..

[22] Nagy J.A., Vasile E., Feng D., Sundberg C., Brown L.F., Detmar M.J., Lawitts J.A., Benjamin L., Tan X., Manseau E.J. (2002). Vascular permeability factor/vascular endothelial growth factor induces lymphangiogenesis as well as angiogenesis. J. Exp. Med..

[23] Liu Y., Cox S.R., Morita T., Kourembanas S. (1995). Hypoxia regulates vascular endothelial growth factor gene expression in endothelial cells. Identification of a 5' enhancer. Circ. Res..

[24] Breier G., Albrecht U., Sterrer S., Risau W. (1992). Expression of vascular endothelial growth factor during embryonic angiogenesis and endothelial cell differentiation. Development.

[25] Ferrara N., Carver-Moore K., Chen H., Dowd M., Lu L., O'Shea K.S., Powell-Braxton L., Hillan K.J., Moore M.W. (1996). Heterozygous embryonic lethality induced by targeted inactivation of the VEGF gene. Nature.

[26] Carmeliet P., Ferreira V., Breier G., Pollefeyt S., Kieckens L., Gertsenstein M., Fahrig M., Vandenhoeck A., Harpal K., Eberhardt C. (1996). Abnormal blood vessel development and lethality in embryos lacking a single VEGF allele. Nature.

[27] Zhu J., Motejlek K., Wang D., Zang K., Schmidt A., Reichardt L.F. (2002). beta8 integrins are required for vascular morphogenesis in mouse embryos. Development.

[28] Shalaby F., Ho J., Stanford W.L., Fischer K.D., Schuh A.C., Schwartz L., Bernstein A., Rossant J. (1997). A requirement for Flk1 in primitive and definitive hematopoiesis and vasculogenesis. Cell.

[29] Risau W. (1997). Mechanisms of angiogenesis. Nature.

[30] Udan R.S., Culver J.C., Dickinson M.E. (2013). Understanding vascular development. Wiley Interdiscip. Rev. Dev. Biol..

[31] Drake C.J., Fleming P.A. (2000). Vasculogenesis in the day 6.5 to 9.5 mouse embryo. Blood.

[32] de Bruijn M.F., Speck N.A., Peeters M.C., Dzierzak E. (2000). Definitive hematopoietic stem cells first develop within the major arterial regions of the mouse embryo. EMBO J..

[33] Inman K.E., Downs K.M. (2007). The murine allantois: Emerging paradigms in development of the mammalian umbilical cord and its relation to the fetus. Genesis.

[34] Fong G.H., Rossant J., Gertsenstein M., Breitman M.L. (1995). Role of the Flt-1 receptor tyrosine kinase in regulating the assembly of vascular endothelium. Nature.

[35] Shalaby F., Rossant J., Yamaguchi T.P., Gertsenstein M., Wu X.F., Breitman M.L., Schuh A.C. (1995). Failure of blood-island formation and vasculogenesis in Flk-1-deficient mice. Nature.

[36] Koch S., Claesson-Welsh L. (2012). Signal transduction by vascular endothelial growth factor receptors. Cold Spring Harb. Perspect. Med..

[37] Hellstrom M., Kalen M., Lindahl P., Abramsson A., Betsholtz C. (1999). Role of PDGF-B and PDGFR-beta in recruitment of vascular smooth muscle cells and pericytes during embryonic blood vessel formation in the mouse. Development.

[38] Heldin C.H., Wasteson A., Westermark B. (1985). Platelet-derived growth factor. Mol. Cell Endocrinol..

[39] Liao H.J., Kume T., McKay C., Xu M.J., Ihle J.N., Carpenter G. (2002). Absence of erythrogenesis and vasculogenesis in Plcg1-deficient mice. J. Biol. Chem..

[40] Wilson R., McGuire C., Mohun T., Project D. (2016). Deciphering the mechanisms of developmental disorders: Phenotype analysis of embryos from mutant mouse lines. Nucleic Acids Res..

[41] Spyropoulos D.D., Pharr P.N., Lavenburg K.R., Jackers P., Papas T.S., Ogawa M., Watson D.K. (2000). Hemorrhage, impaired hematopoiesis, and lethality in mouse embryos carrying a targeted disruption of the Fli1 transcription factor. Mol. Cell Biol..

[42] McCarty J.H., Monahan-Earley R.A., Brown L.F., Keller M., Gerhardt H., Rubin K., Shani M., Dvorak H.F., Wolburg H., Bader B.L. (2002). Defective associations between blood vessels and brain parenchyma lead to cerebral hemorrhage in mice lacking alphav integrins. Mol. Cell Biol..

[43] Gupta A., Rarick K.R., Ramchandran R. (2021). Established, New and Emerging Concepts in Brain Vascular Development. Front. Physiol..

