# Supplementary material for: Transmembrane-4 L-Six Family Member-1 Is Essential for Embryonic Blood Vessel Development
Source: Curr Issues Mol Biol. 2024 Nov 18;46(11):13105–18. doi: 10.3390/cimb46110781 (PMC11592815; doi:10.3390/cimb46110781)
Supplement: Supplementary file 1 [file cimb-46-00781-s001.zip › Supplemental Figures, Movies & Tables.pptx]

## Slide 1
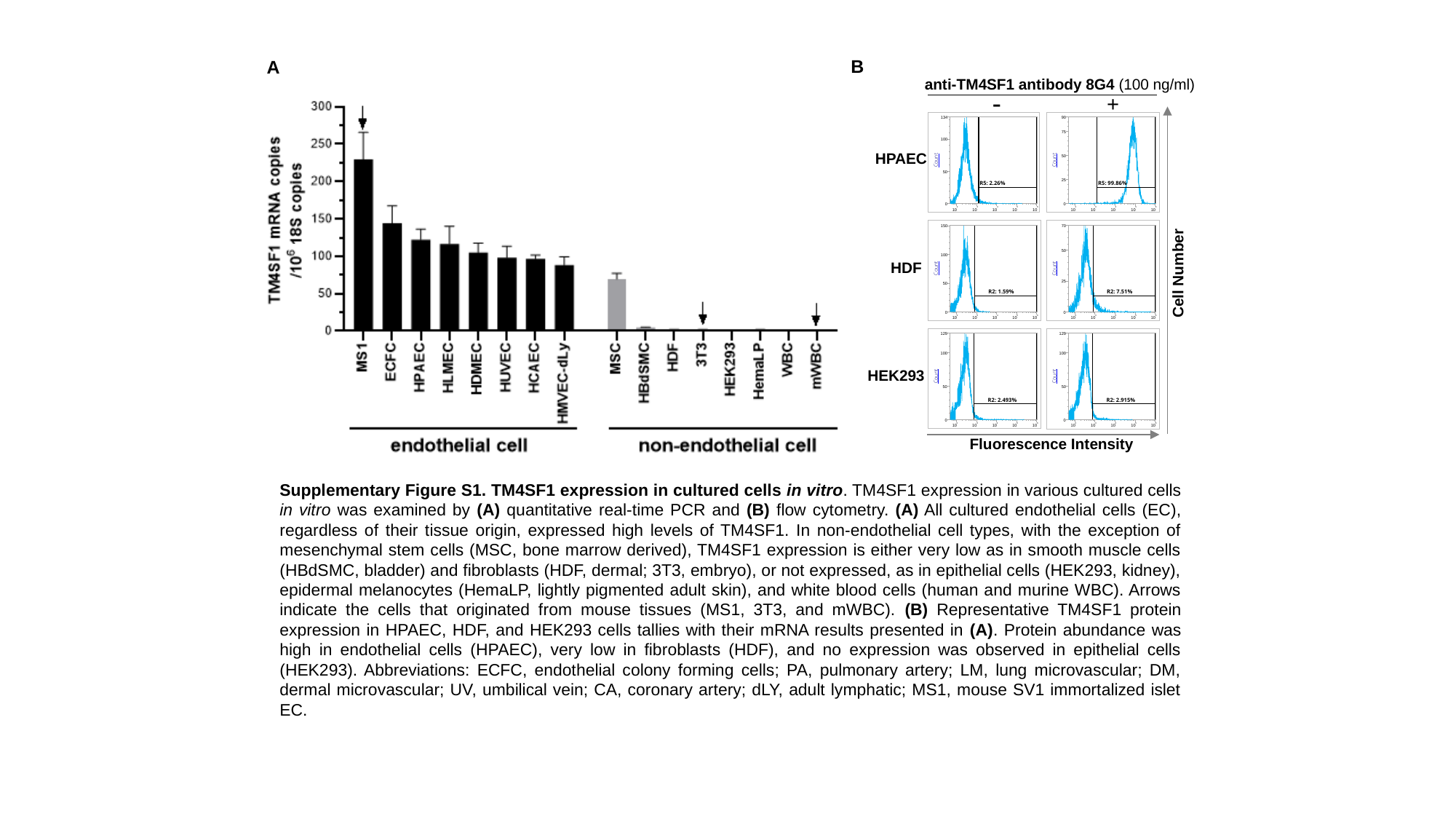

B
anti-TM4SF1 antibody 8G4 (100 ng/ml)
-
+
HPAEC
HDF
Cell Number
HEK293
Fluorescence Intensity
A
Supplementary Figure S1. TM4SF1 expression in cultured cells in vitro. TM4SF1 expression in various cultured cells in vitro was examined by (A) quantitative real-time PCR and (B) flow cytometry. (A) All cultured endothelial cells (EC), regardless of their tissue origin, expressed high levels of TM4SF1. In non-endothelial cell types, with the exception of mesenchymal stem cells (MSC, bone marrow derived), TM4SF1 expression is either very low as in smooth muscle cells (HBdSMC, bladder) and fibroblasts (HDF, dermal; 3T3, embryo), or not expressed, as in epithelial cells (HEK293, kidney), epidermal melanocytes (HemaLP, lightly pigmented adult skin), and white blood cells (human and murine WBC). Arrows indicate the cells that originated from mouse tissues (MS1, 3T3, and mWBC). (B) Representative TM4SF1 protein expression in HPAEC, HDF, and HEK293 cells tallies with their mRNA results presented in (A). Protein abundance was high in endothelial cells (HPAEC), very low in fibroblasts (HDF), and no expression was observed in epithelial cells (HEK293). Abbreviations: ECFC, endothelial colony forming cells; PA, pulmonary artery; LM, lung microvascular; DM, dermal microvascular; UV, umbilical vein; CA, coronary artery; dLY, adult lymphatic; MS1, mouse SV1 immortalized islet EC.

## Slide 2
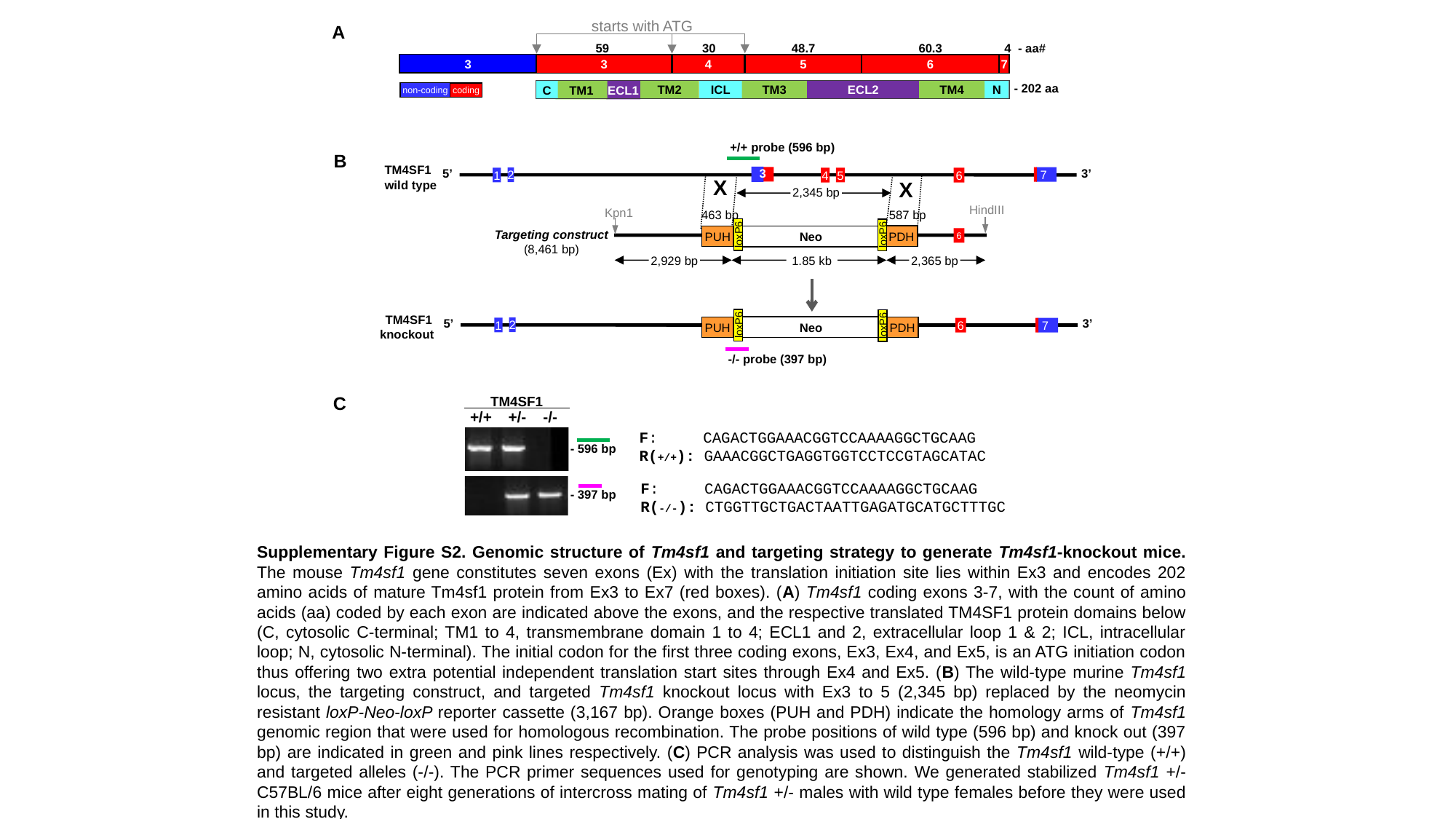

starts with ATG
 59 30 48.7 60.3 4 - aa#
3
7
3
4
5
6
- 202 aa
ECL2
TM4
N
TM2
ICL
TM3
C
TM1
ECL1
non-coding
coding
A
+/+ probe (596 bp)
TM4SF1
wild type
7
2
5
1
4
6
5’
3’
3
X
X
2,345 bp
HindIII
Kpn1
463 bp
587 bp
Targeting construct (8,461 bp)
PDH
Neo
PUH
6
loxP6
loxP6
2,929 bp
1.85 kb
2,365 bp
TM4SF1 knockout
5’
3’
Neo
PDH
PUH
2
1
6
7
loxP6
loxP6
-/- probe (397 bp)
B
C
TM4SF1
+/+ +/- -/-
F: CAGACTGGAAACGGTCCAAAAGGCTGCAAG
R(+/+): GAAACGGCTGAGGTGGTCCTCCGTAGCATAC
- 596 bp
F: CAGACTGGAAACGGTCCAAAAGGCTGCAAG
R(-/-): CTGGTTGCTGACTAATTGAGATGCATGCTTTGC
- 397 bp
Supplementary Figure S2. Genomic structure of Tm4sf1 and targeting strategy to generate Tm4sf1-knockout mice. The mouse Tm4sf1 gene constitutes seven exons (Ex) with the translation initiation site lies within Ex3 and encodes 202 amino acids of mature Tm4sf1 protein from Ex3 to Ex7 (red boxes). (A) Tm4sf1 coding exons 3-7, with the count of amino acids (aa) coded by each exon are indicated above the exons, and the respective translated TM4SF1 protein domains below (C, cytosolic C-terminal; TM1 to 4, transmembrane domain 1 to 4; ECL1 and 2, extracellular loop 1 & 2; ICL, intracellular loop; N, cytosolic N-terminal). The initial codon for the first three coding exons, Ex3, Ex4, and Ex5, is an ATG initiation codon thus offering two extra potential independent translation start sites through Ex4 and Ex5. (B) The wild-type murine Tm4sf1 locus, the targeting construct, and targeted Tm4sf1 knockout locus with Ex3 to 5 (2,345 bp) replaced by the neomycin resistant loxP-Neo-loxP reporter cassette (3,167 bp). Orange boxes (PUH and PDH) indicate the homology arms of Tm4sf1 genomic region that were used for homologous recombination. The probe positions of wild type (596 bp) and knock out (397 bp) are indicated in green and pink lines respectively. (C) PCR analysis was used to distinguish the Tm4sf1 wild-type (+/+) and targeted alleles (-/-). The PCR primer sequences used for genotyping are shown. We generated stabilized Tm4sf1 +/- C57BL/6 mice after eight generations of intercross mating of Tm4sf1 +/- males with wild type females before they were used in this study.

## Slide 3
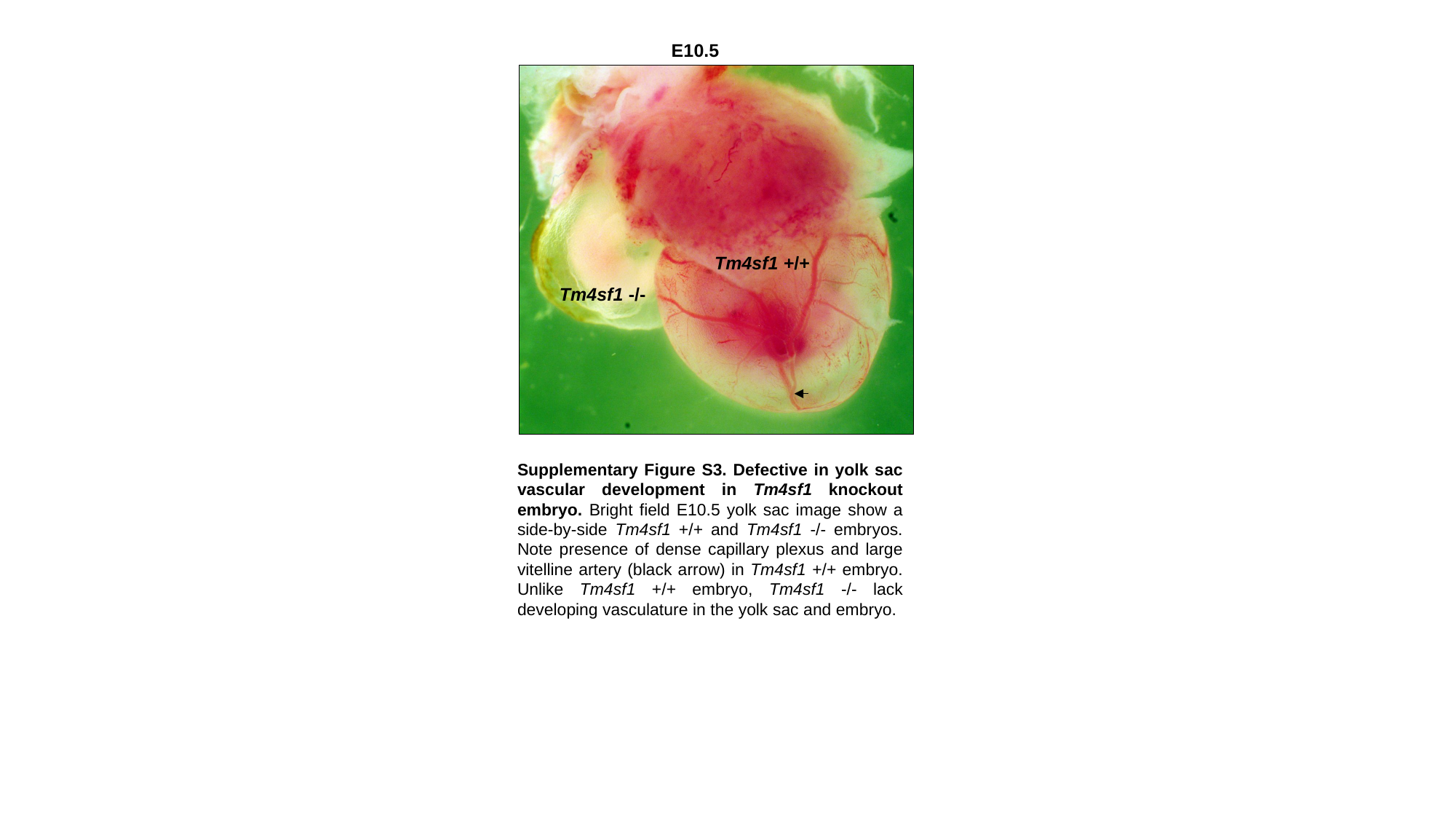

E10.5
Tm4sf1 +/+
Tm4sf1 -/-
Supplementary Figure S3. Defective in yolk sac vascular development in Tm4sf1 knockout embryo. Bright field E10.5 yolk sac image show a side-by-side Tm4sf1 +/+ and Tm4sf1 -/- embryos. Note presence of dense capillary plexus and large vitelline artery (black arrow) in Tm4sf1 +/+ embryo. Unlike Tm4sf1 +/+ embryo, Tm4sf1 -/- lack developing vasculature in the yolk sac and embryo.

## Slide 4
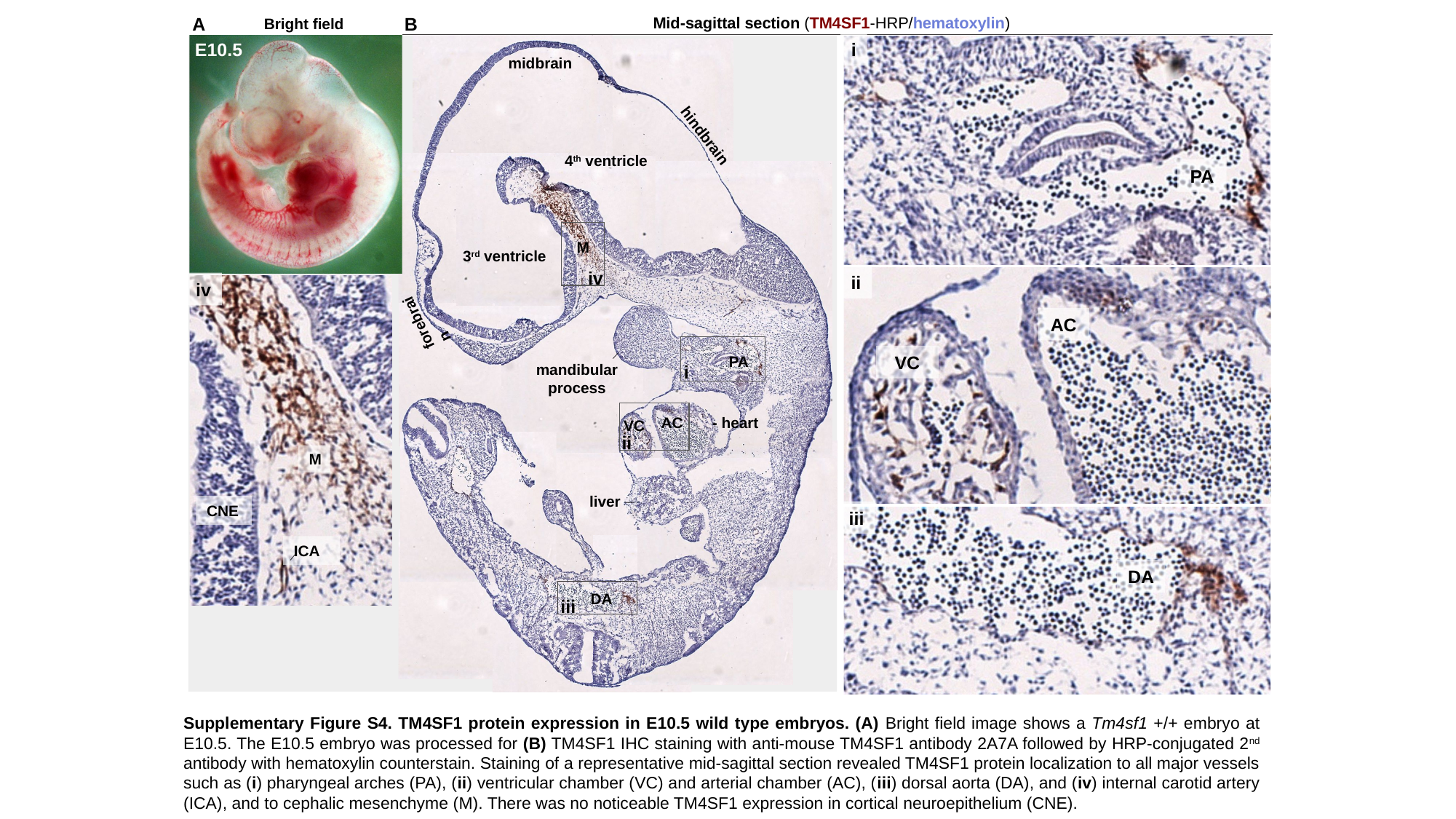

A
B
Mid-sagittal section (TM4SF1-HRP/hematoxylin)
Bright field
E10.5
i
midbrain
hindbrain
4th ventricle
PA
M
3rd ventricle
iv
ii
iv
AC
forebrain
_
mandibular process
VC
PA
i
- heart
AC
VC
ii
M
_
liver
CNE
iii
ICA
_
DA
DA
iii
Supplementary Figure S4. TM4SF1 protein expression in E10.5 wild type embryos. (A) Bright field image shows a Tm4sf1 +/+ embryo at E10.5. The E10.5 embryo was processed for (B) TM4SF1 IHC staining with anti-mouse TM4SF1 antibody 2A7A followed by HRP-conjugated 2nd antibody with hematoxylin counterstain. Staining of a representative mid-sagittal section revealed TM4SF1 protein localization to all major vessels such as (i) pharyngeal arches (PA), (ii) ventricular chamber (VC) and arterial chamber (AC), (iii) dorsal aorta (DA), and (iv) internal carotid artery (ICA), and to cephalic mesenchyme (M). There was no noticeable TM4SF1 expression in cortical neuroepithelium (CNE).

## Slide 5
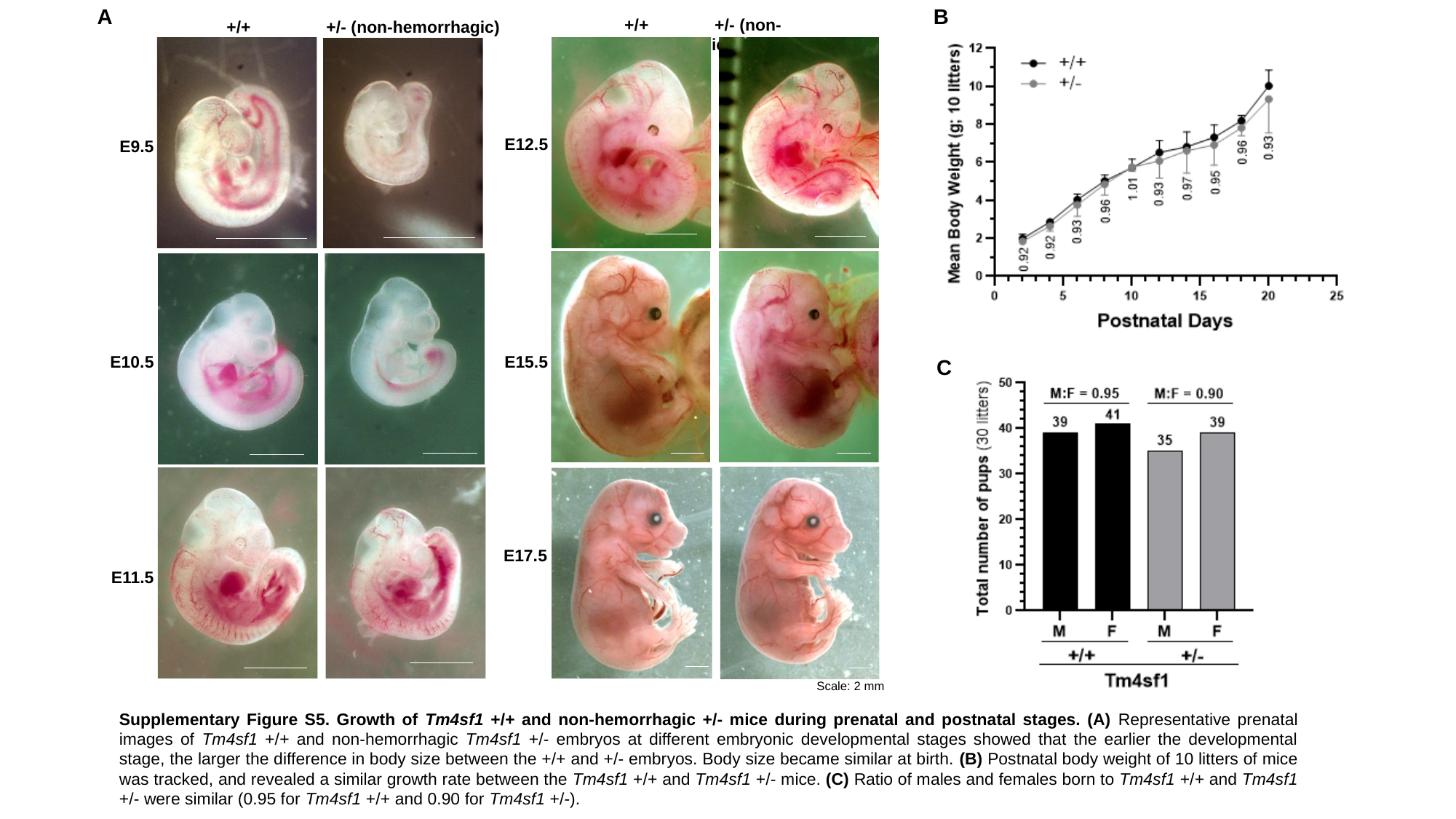

A
B
+/+ +/- (non-hemorrhagic)
E12.5
E15.5
E17.5
Scale: 2 mm
+/+ +/- (non-hemorrhagic)
E9.5
E10.5
E11.5
C
Supplementary Figure S5. Growth of Tm4sf1 +/+ and non-hemorrhagic +/- mice during prenatal and postnatal stages. (A) Representative prenatal images of Tm4sf1 +/+ and non-hemorrhagic Tm4sf1 +/- embryos at different embryonic developmental stages showed that the earlier the developmental stage, the larger the difference in body size between the +/+ and +/- embryos. Body size became similar at birth. (B) Postnatal body weight of 10 litters of mice was tracked, and revealed a similar growth rate between the Tm4sf1 +/+ and Tm4sf1 +/- mice. (C) Ratio of males and females born to Tm4sf1 +/+ and Tm4sf1 +/- were similar (0.95 for Tm4sf1 +/+ and 0.90 for Tm4sf1 +/-).

## Slide 6
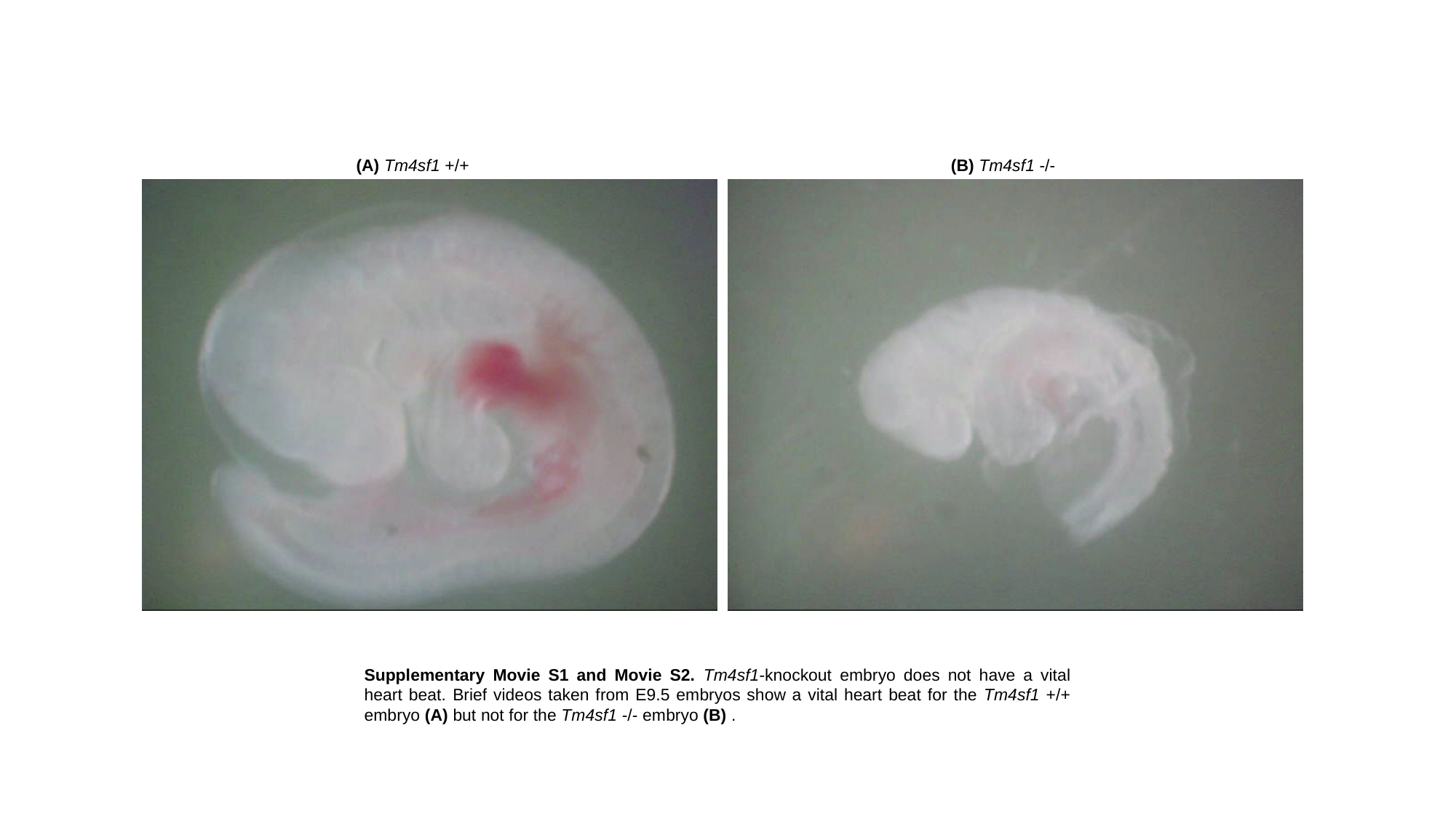

(A) Tm4sf1 +/+
(B) Tm4sf1 -/-
Supplementary Movie S1 and Movie S2. Tm4sf1-knockout embryo does not have a vital heart beat. Brief videos taken from E9.5 embryos show a vital heart beat for the Tm4sf1 +/+ embryo (A) but not for the Tm4sf1 -/- embryo (B) .

## Slide 7
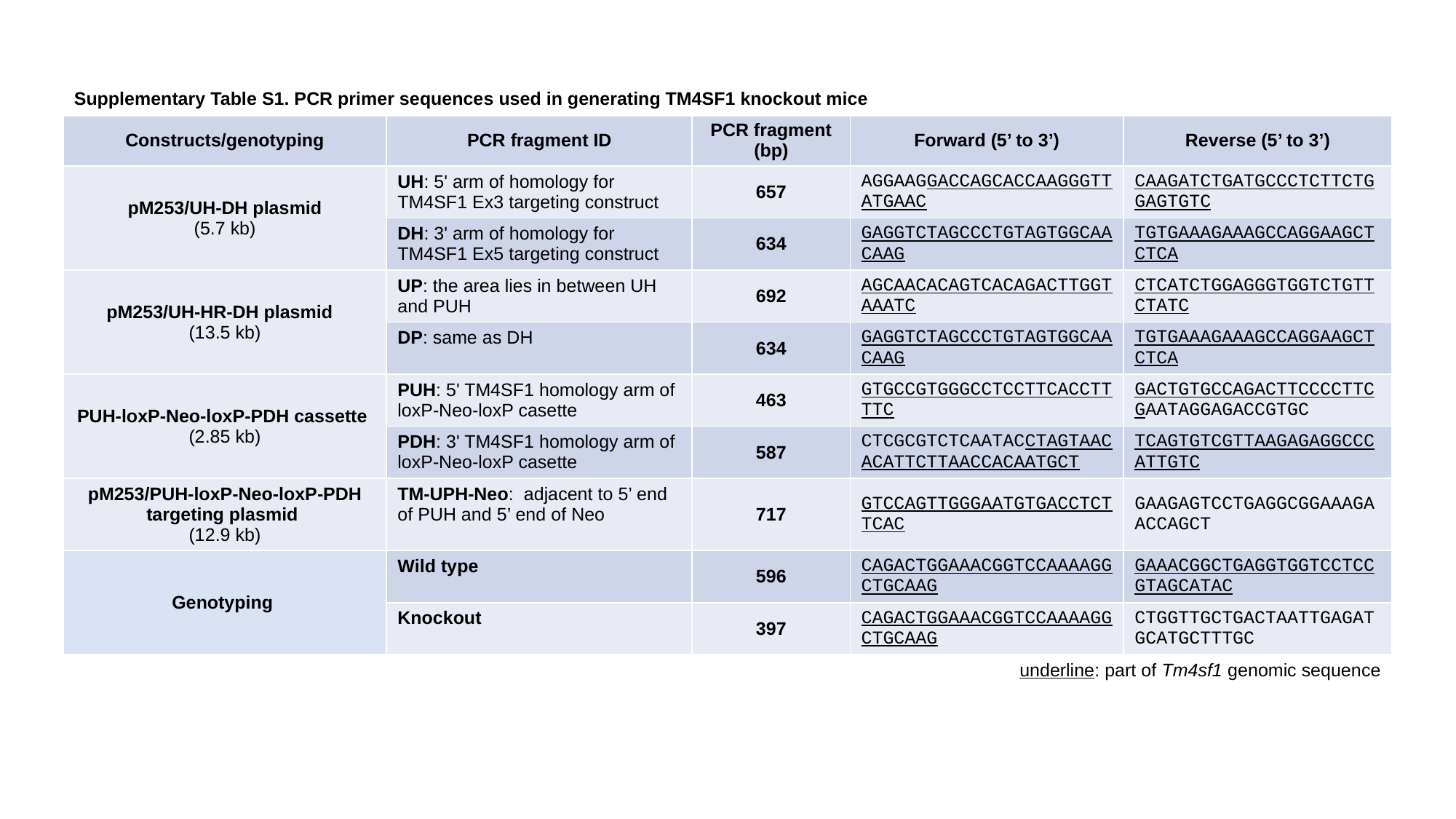

| Supplementary Table S1. PCR primer sequences used in generating TM4SF1 knockout mice | | | | |
| --- | --- | --- | --- | --- |
| Constructs/genotyping | PCR fragment ID | PCR fragment (bp) | Forward (5’ to 3’) | Reverse (5’ to 3’) |
| pM253/UH-DH plasmid (5.7 kb) | UH: 5' arm of homology for TM4SF1 Ex3 targeting construct | 657 | AGGAAGGACCAGCACCAAGGGTTATGAAC | CAAGATCTGATGCCCTCTTCTGGAGTGTC |
| | DH: 3' arm of homology for TM4SF1 Ex5 targeting construct | 634 | GAGGTCTAGCCCTGTAGTGGCAACAAG | TGTGAAAGAAAGCCAGGAAGCTCTCA |
| pM253/UH-HR-DH plasmid (13.5 kb) | UP: the area lies in between UH and PUH | 692 | AGCAACACAGTCACAGACTTGGTAAATC | CTCATCTGGAGGGTGGTCTGTTCTATC |
| | DP: same as DH | 634 | GAGGTCTAGCCCTGTAGTGGCAACAAG | TGTGAAAGAAAGCCAGGAAGCTCTCA |
| PUH-loxP-Neo-loxP-PDH cassette (2.85 kb) | PUH: 5' TM4SF1 homology arm of loxP-Neo-loxP casette | 463 | GTGCCGTGGGCCTCCTTCACCTTTTC | GACTGTGCCAGACTTCCCCTTCGAATAGGAGACCGTGC |
| | PDH: 3' TM4SF1 homology arm of loxP-Neo-loxP casette | 587 | CTCGCGTCTCAATACCTAGTAACACATTCTTAACCACAATGCT | TCAGTGTCGTTAAGAGAGGCCCATTGTC |
| pM253/PUH-loxP-Neo-loxP-PDH targeting plasmid (12.9 kb) | TM-UPH-Neo: adjacent to 5’ end of PUH and 5’ end of Neo | 717 | GTCCAGTTGGGAATGTGACCTCTTCAC | GAAGAGTCCTGAGGCGGAAAGAACCAGCT |
| Genotyping | Wild type | 596 | CAGACTGGAAACGGTCCAAAAGGCTGCAAG | GAAACGGCTGAGGTGGTCCTCCGTAGCATAC |
| | Knockout | 397 | CAGACTGGAAACGGTCCAAAAGGCTGCAAG | CTGGTTGCTGACTAATTGAGATGCATGCTTTGC |
| underline: part of Tm4sf1 genomic sequence | | | | |

## Slide 8
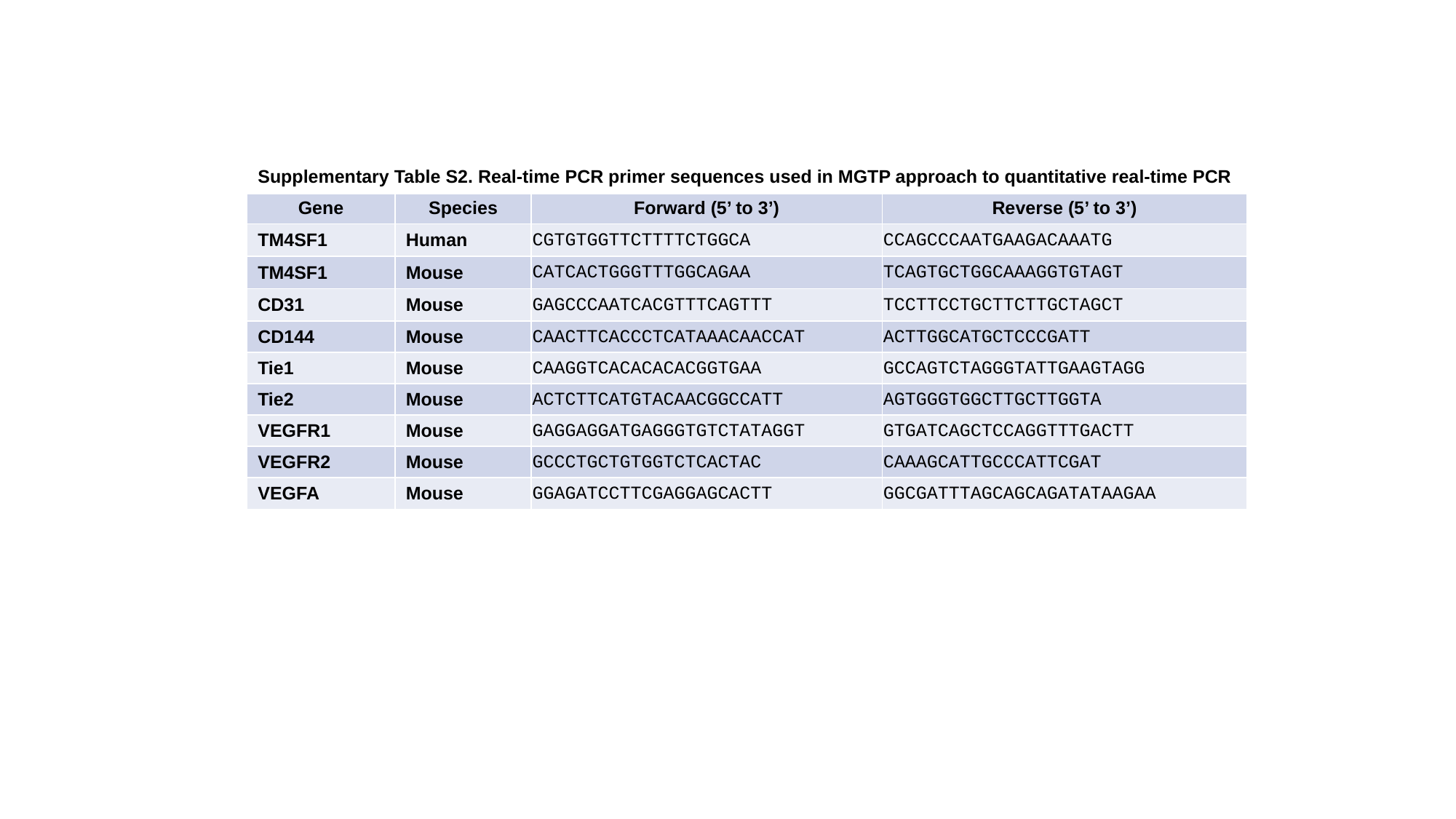

| Supplementary Table S2. Real-time PCR primer sequences used in MGTP approach to quantitative real-time PCR | | | | |
| --- | --- | --- | --- | --- |
| Gene | | Species | Forward (5’ to 3’) | Reverse (5’ to 3’) |
| TM4SF1 | | Human | CGTGTGGTTCTTTTCTGGCA | CCAGCCCAATGAAGACAAATG |
| TM4SF1 | | Mouse | CATCACTGGGTTTGGCAGAA | TCAGTGCTGGCAAAGGTGTAGT |
| CD31 | | Mouse | GAGCCCAATCACGTTTCAGTTT | TCCTTCCTGCTTCTTGCTAGCT |
| CD144 | | Mouse | CAACTTCACCCTCATAAACAACCAT | ACTTGGCATGCTCCCGATT |
| Tie1 | | Mouse | CAAGGTCACACACACGGTGAA | GCCAGTCTAGGGTATTGAAGTAGG |
| Tie2 | | Mouse | ACTCTTCATGTACAACGGCCATT | AGTGGGTGGCTTGCTTGGTA |
| VEGFR1 | | Mouse | GAGGAGGATGAGGGTGTCTATAGGT | GTGATCAGCTCCAGGTTTGACTT |
| VEGFR2 | | Mouse | GCCCTGCTGTGGTCTCACTAC | CAAAGCATTGCCCATTCGAT |
| VEGFA | | Mouse | GGAGATCCTTCGAGGAGCACTT | GGCGATTTAGCAGCAGATATAAGAA |
| | | | | |
